# Whole-cell bioreporter technology: a promising approach for environmental risk assessment of As contamination in soil

**DOI:** 10.3389/fmicb.2024.1494872

**Published:** 2024-11-21

**Authors:** Xiaokai Zhang, Xinyu Zhao, Caiwen Gu, Zefeng Huang, Tao Gan, Boling Li, Evrim Elçin, Lizhi He

**Affiliations:** ^1^School of Environment and Ecology, Institute of Environmental Processes and Pollution Control, Jiangnan University, Wuxi, China; ^2^School of Environmental Science and Engineering, Suzhou University of Science and Technology, Suzhou, Jiangsu, China; ^3^Meadows Center for Water and the Environment, Texas State University, San Marcos, TX, United States; ^4^Division of Enzyme and Microbial Biotechnology, Department of Agricultural Biotechnology, Faculty of Agriculture, Aydın Adnan Menderes University, Aydın, Türkiye; ^5^College of Environmental and Resource Sciences, Zhejiang A&F University, Hangzhou, China

**Keywords:** biosensor, soil, arsenic, environmental risk assessment, bioavailability, heavy metal

## 1 Introduction

Arsenic (As) is an extremely toxic metalloid that has attracted considerable attention worldwide due to its harmful effects on humans, animals and plants. It is estimated that millions of people around the world at risk of being affected by As contamination (Rahman et al., 2017). As species mainly enters the soil through different routes, including surface runoff of industrial As-containing wastewater, the application of pesticides containing As, chemical fertilizers, organic fertilizers and other human activities (Boulanger et al., 2019). In addition, natural factors such as volcanic movement, flooding, chemical weathering also contribute to excessive As levels in the soil (Huq et al., 2020). The As that enter the soil can accumulate in crops and enter the human body through the food chain, leading to the development of diseases including skin, lung and bladder cancer (Palansooriya et al., 2020; Palma-Lara et al., 2020).

As in the environment can be found in both inorganic and organic forms, while the inorganic forms, including arsenate, As(V) and arsenite, As(III), are the predominant forms in soils, and studies have found that As(III) has greater mobility and greater toxicity than As(V) (Gao et al., 2022; Gorny et al., 2015; Lee et al., 2019). Changes in the form and valence of As in soil directly affect its bioavailability and toxicity, and thus its environmental risk. Therefore, it is important to conduct environmental risk assessment of As in soil to safeguard soil health. Two commonly used approaches of environmental risk assessment include the ground accumulation index method and potential ecological risk assessment method. However, they all based on the detection of concentrations of As by traditional analytical methods such as inductively coupled plasma emission spectrometry (ICP-OES) (Henry and Thorpe, 1980) and atomic absorption spectrometry (AAS) (Aggett and Aspell, 1976). These methods usually require expensive instruments and specialized personnel to pre-treat and analyze the samples. In addition, they are unable to reflect the bioavailability of As (Bereza-Malcolm et al., 2015). Therefore, streamlining this risk assessment process is essential for the rapid identification of environmental risks of As in soil.

In recent years, whole-cell bioreporter (WCB) technology has been used for the assessment of the bioavailability of heavy metals in the environment and consequently to evaluate their environmental risks (Huang et al., 2024). WCB is a living genetically engineered bacterial, which can sense target chemicals and generate electrochemical or optical signals that can be detected, and determine the bioavailability or toxicity of pollutants (Dhyani et al., 2022). In addition, it has been reported that WCB can both assess the bioavailability of As in the environment and differentiate the different forms of As (Yoon et al., 2016b). The application of WCB technology, as reviewed herein, represents a progressive shift toward more sensitive, cost-effective, and ecologically relevant environmental risk assessment.

## 2 Construction principles of WCB for detecting As bioavailability

**Figure 1:**
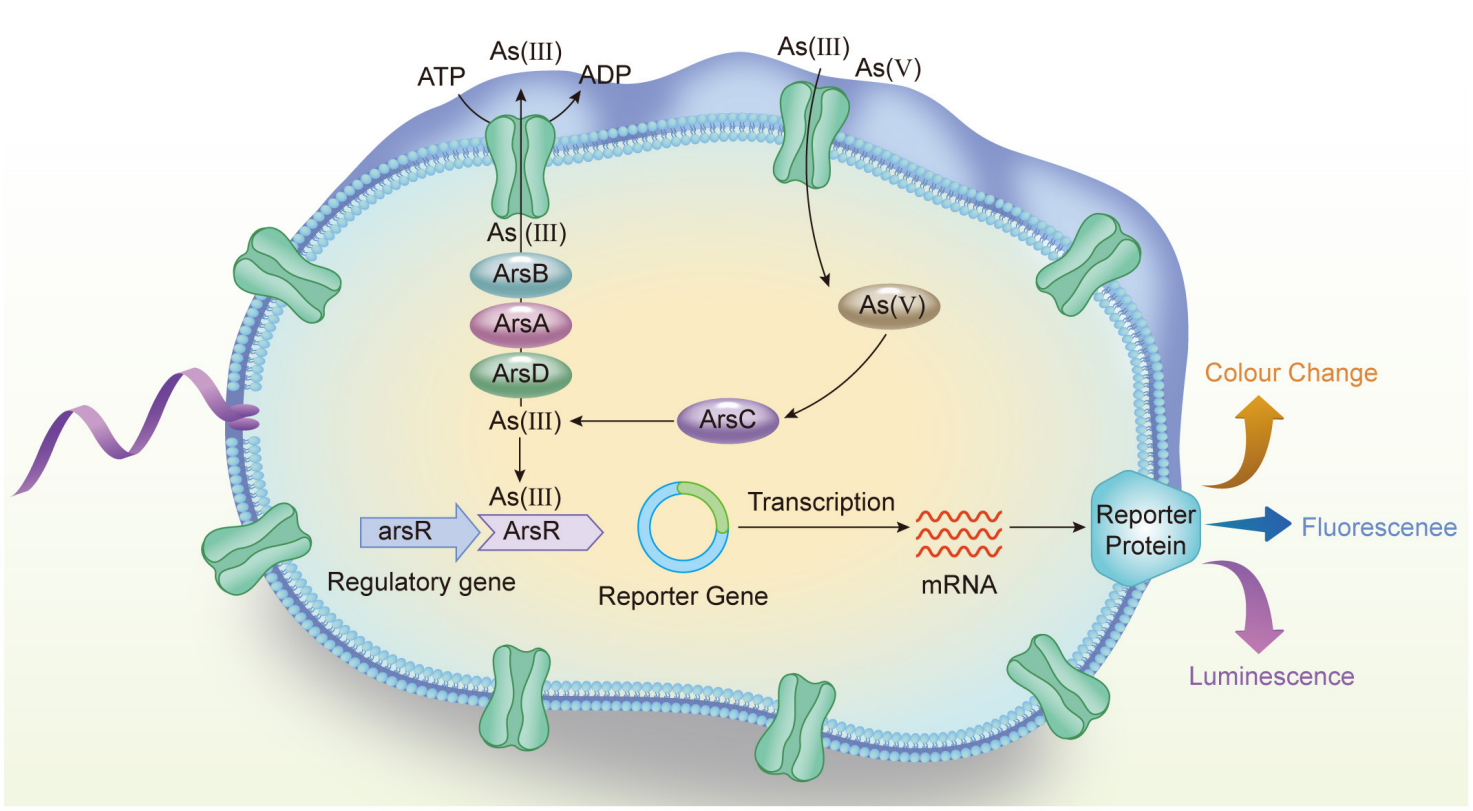
Schematic diagram of whole-cell bioreporter for As detection.

WCB technology, by harnessing the biological responses of microbial systems, offers a direct measure of As bioavailability and its potential risks. Generally, As-WCBs were constructed based on sensing element from As-resistance operon *ars* and reporter genes (Kaur et al., 2015). Bacteria such as *E. coli, Pseudomonas*, and *Staphylococcus aureus* are often selected as host strains of As-WCB because they carry genes with specific resistance mechanisms to As, exhibiting As resistance (Bereza-Malcolm et al., 2018). *ars* operon has been found in above-mentioned bacteria and known as the optimal microbial As detoxification system (Ordóñez et al., 2005). Hedges and Baumberg (1973) and Mobley et al. (1983) found that the plasmid R773 in *Escherichia coli* (*E. coli*) can help strains acquire As resistance, and further research on the R773 plasmid revealed the existence of a gene cluster, the *ars* operon, conferring As resistance. *ars* operon contains five co-transcribed genes (*arsRDABC*), *arsR* is identified as As regulatory gene, and the encodes regulatory protein ArsR which controls the basal expression level ensuring that the expression level of As resistance operon in different environments is within a certain range (Arik et al., 2023). Since it plays an important role in regulation of intracellular As levels, ArsR regulatory protein from bacterial origin has been often deployed in WCB technology for determination of environmental As contamination. When the sample does not contain As(III), ArsR binds to the binding site (ABS) of the promoter of *ars* operon (P_ars_R), inhibiting its expression (Valenzuela-García et al., 2023). Reversely, in the presence of As(III), As(III) binds to ArsR and changes its conformation, relieving ArsR inhibition of P_ars_R, which is shown in Figure 1 (Yoon et al., 2016b). The ArsRBC proteins in the *ars* operon regulated by the ArsR protein cooperate to form a regulatory mechanism for As transport and maintain the balance of As in the cell. Although ArsR only responds to As(III), As-WCB can also respond to As(V) because sensing strains containing chromosome-encoded *arsRBC* operons produce moderate resistance to As and arsenate is reduced to arsenite in bacterial cells (Elcin and Öktem, 2019). Apart from ArsR, ArsC reduces As(V) to As(III), ArsB excretes As(III) from cells using the potential drive on the cell membrane, and the structural gene *arsA*, which codes for the As ATPase subunit ArsA, and the regulatory gene *arsD*, which codes for the arsenite chaperone that transports As(III) to the ArsAB transporter complex (Irvine et al., 2017; Yang et al., 2011; Yu et al., 2021).

In addition to the *ars* operon, As-WCB developed using the nikA promoter of the *nik* operon in *E. coli* also specifically detects As. Similar to the mechanism of action of the *ars* operon, As(III) interacts with residues (His79 and His92) on NikR, and the conformational change in NikR may lead to its release from the promoter region of the *nik* operon, which would increase the transcription of the reporter gene for the detection of As(III) in soil (Yoon et al., 2016a).

Since most of the As sensing WCB are based on bacterial cells, the type, culture, and number of host bacteria, as well as the growth status of bacteria, may affect the detection performance of WCB. For example, Wu et al. (2023) discovered that the optimal assay stability was attained when WCB was incubated at 30°C until the exponential growth period and that selecting a sample volume of 200 μl produced the highest luminescence or fluorescence output. Therefore, on the basis of extensive research on As-WCB construction, optimizing the detection environment and promoting its practical applications will be the main research target in this field.

## 3 Application of WCBs in assessing As bioavailability

Cai and DuBow (1997) constructed the first WCB based on the fusion of an As resistance operon and luciferase gene in *E. coli* for the detection of chromate arsenate, which provided a good research direction for the detection of As toxicity by WCB. Over the next two decades, to enhance the sensitivity, specificity and stability of As detection, researchers have developed the As-WCB using different sensing and reporter elements. A novel As-WCB constructed by fusing the *nikA* gene from *nik* operon in *E. coli* and the green fluorescent protein encoding gene which selective only for As among eight metals has been used for the determination of As in soil (Yoon et al., 2016a). Pola-López et al. (2018) amplified the As input signal by adding a T7 RNAP gene amplifier module within the strain, significantly enhanced the sensitivity performance of the WCB and successfully detected arsenate in the range of 5–140 μg/L with a response time as little as an hour. Chen et al. (2022) combined an As-WCB based on the optimized P_arsOC2_ promoter with a smartphone color recognition application to analyze As in groundwater samples with a detection limit as low as 0.24 μg/L.

It is worth noting that As in the natural environment usually exists in the form of As(III) or As(V), which are different in toxicity [As(III) possesses a toxicity 60 times greater than As(V)]. They can be easily transformed into each other when there is a change in the redox potential, pH, and oxygen-enriched state, which poses a great challenge to accurate assess the environmental risk of As in different valence states (Pena et al., 2005). In a recent study, Elcin and Öktem (2019) constructed As-WCB on the basis of *arsR* regulatory gene of *E. coli* plasmid R773 and a green fluorescent reporter protein using *E. coli* MG1655 host strain. The WCB can specifically assess both As(III) and As(V) in phosphate-restricted medium and to differentiate between the two on the basis of the response time at 10 ppb level.

However, no matter how sophisticated genetic engineering techniques are used, they can only be applied in the field outside the laboratory if the WCBs are secured into a suitable platform and become portable sensing devices. For this purpose, immobilization methods that have been developed for WCB include lyophilization (Bilal and Iqbal, 2019), agar (Huang et al., 2024) and calcium alginate entrapment (Tohfegar and Habibi, 2023), and embedded fiber-optics (Zhu et al., 2022). Elcin and Öktem (2020) immobilized the As-WCB cells into agar hydrogels and alginate beads for a first-step field application and reported that under optimal incubation conditions, it could detect 10 μg/L and 200 μg/L of As(III) and As(V) within 5 hour and 2 hour, respectively. In addition, it was found in the experiment that adjusting the cell density of OD_600_ to 0.4 significantly improved the sensitivity of WCB. Arik et al. (2023) used polycaprolactone (PCL) electrostatically spun fibers as a support material to immobilize WCB, and found that the system was able to be used in natural waters and sensitive only to As which can rapidly detect As(III) in the range of 10–100 μg/L.

In addition, the coupling of WCB with other chemical methods provides new ideas for the field application of WCB. Buffi et al. (2011) attempted to immobilize WCB in agarose beads and integrate them into a microfluidic chip for on-site monitoring of As. Their study showed that the strain maintained performance for up to 1 month when stored in the microfluidic chip at −20°C and was able to respond to arsenate within a concentration bracket of 10–50 μg/L. In a recent study, Sánchez et al. (2021) combined As-WCB with electrochemical measurements, strains produced electrochemically detectable 4-aminophenol in the presence of As(III). The system provides higher accuracy and signal strength than traditional WCB detection methods and has garnered regulatory clearance for field use in both Canada and the United States. Soil systems are more complex than water environment, research on As-WCB for soil on-site measurement is relatively immature. Due to the attenuation of the WCB signal by soil particles, the transmission of the optical signal from the WCB is reduced, thus affecting the accuracy of the results. It has been reported that many studies have neglected the weakening of the WCB signal by soil, which can lead to orders of magnitude errors in the results (Zhang et al., 2022). To date, there is no standard to unify the method, which has brought some challenges for the application of As-WCB in soil. However, in view of the intensity of As contamination in soil, we believe that the development of portable, low-cost, and highly sensitive *in situ* WCB for detecting As bioavailability in soil will be a future direction.

## 4 Conclusions

The environmental contamination of As is highly complex and its toxicity varies according to the different forms present in the soil, making it particularly important to accurately assess and differentiate the bioavailability of these forms of As. WCB reflects the bioavailability of the As and has the potential to discriminate between different forms of As. As yet, WCB has not yet become a standard method for environmental risk assessment of As contamination, and only a small number of WCB has been commercialized. We believe that with continuous and in-depth development, WCB is expected to become an effective environmental risk assessment method for As contamination in soil. As we advance, it becomes crucial to not only understand but also anticipate the ecological impacts of contaminants. WCB technology emerges as a critical tool in this regard, facilitating a more proactive approach to environmental management.
